# Transmembrane Protein 170B is a Prognostic Biomarker and Associated With Immune Infiltrates in Pancreatic Adenocarcinoma

**DOI:** 10.3389/fgene.2022.848391

**Published:** 2022-05-05

**Authors:** Zilong Zhang, Jin Shang, Zonglin Dai, Yutong Yao, Ying Shi, Deyuan Zhong, Yuxin Liang, Chunyou Lai, Qinyan Yang, Tianhang Feng, Xiaolun Huang

**Affiliations:** ^1^ Department of Hepatobiliary-Pancreatic Surgery, Cell Transplantation Center, Sichuan Provincial People’s Hospital, University of Electronic Science and Technology of China, Chengdu, China; ^2^ Chinese Academy of Sciences Sichuan Translational Medicine Research Hospital, Chengdu, China; ^3^ School of Medicine, University of Electronic Science and Technology of China, Chengdu, China

**Keywords:** TMEM170B, prognosis, pancreatic cancer, biomarker, immune infiltration

## Abstract

**Background:** Pancreatic adenocarcinoma (PAAD) is among the most common types of cancer with a poor prognosis. Transmembrane protein 170B (TMEM170B) has been reported to suppress breast cancer proliferation, metastasis, and tumorigenesis and is related to prognosis. However, its role in PAAD and the underlying molecular mechanisms are yet to be investigated.

**Patients and methods:** We performed a comprehensive analysis of RNA sequencing data obtained from the Gene Expression Omnibus (GEO) and The Cancer Genome Atlas (TCGA) databases to determine TMEM170B expression. Immunostaining and real-time polymerase chain reaction (RT-PCR) were done to determine TMEM170B expression in human pancreatic cancer cell lines and tissue specimens. Furthermore, the correlation of TMEM170B with clinicopathological features and PAAD prognosis was investigated, and the mechanisms were explored through enrichment analysis and immune cell infiltration analysis.

**Results:** TCGA and GEO dataset analysis revealed that TMEM170B expression in PAAD tissue samples was significantly lower than that in non-tumorous tissues, which was further confirmed by immunohistochemistry and RT-PCR. Low TMEM170B expression was associated with poor differentiation (*p* = 0.014). Multivariate analysis identified that TMEM170B is an independent indicator for overall survival [hazard ratio (HR) = 0.116, 95% confidence interval (CI) = 0.014–0.995; *p* = 0.049] and disease-free survival (HR = 0.19, 95% CI = 0.04–0.910; *p* = 0.038) in patients with PAAD. Additionally, TMEM170B was involved in immune-related gene sets, including those related to chemokine signaling pathways and innate and adaptive immunity. High TMEM170B expression was linked to antitumor immune microenvironment with a high infiltration of B cells, T cells, dendritic cells, monocytes, M1 macrophages, neutrophil, and natural killer cells and a low infiltration of Tregs and myeloid-derived suppressor cells (all *p* < 0.05).

**Plain Language Summary:** There is an urgent need to identify clinical prognostic biomarkers and targeted drugs for pancreatic cancer treatment. In this study, the expression status and prognostic value of transmembrane protein 170B (TMEM170B) in pancreatic adenocarcinoma were elucidated. Furthermore, TMEM170B, as a tumor suppressor gene, induced antitumor immune effects, including increased tumor infiltration of immune effector cells and reduced levels of inhibitory immune molecules and regulatory cells. Therefore, TMEM170B could be regarded as a novel target in preventing the progression of pancreatic cancer.

**Conclusion:** The findings suggest that low TMEM170B expression is remarkably correlated with poor PAAD prognosis, which might provide a therapeutic target for PAAD.

## 1 Introduction

Pancreatic cancer is a common malignant tumor of the digestive tract and the seventh leading cause of cancer-related death worldwide in 2020, with approximately 4,96,000 newly diagnosed cases and 4,66,000 deaths Sung et al. (2021). Owing to its inapparent symptoms and rapid progression, 80% of patients with pancreatic cancer fail to meet the criteria for radical resection Connor and Gallinger, (2021). Furthermore, the high rates of late recurrence and metastasis and numerous postoperative complications also result in unsatisfying surgical outcomes and poor prognosis. For the reasons mentioned above, pancreatic adenocarcinoma (PAAD) has one of the highest death rates of any solid organ malignancy, with overall 5-years survival of less than 8% Connor and Gallinger, (2021); Di Marco et al. (2016); Siegel et al. (2018). In recent years, significant improvements in molecular diagnosis and targeted biological therapy have facilitated the discovery of novel genetic biomarkers and drug targets. Such advancements have helped in elucidating the molecular mechanisms of pancreatic cancer and have gradually becoming important means for improving the overall prognosis of the patients Cassetta and Kitamura, (2018); Mace et al. (2018); O'Reilly et al. (2019); Waddell et al. (2015). Additionally, tumor-related genes have continuously been found to be associated with the incidence and development of pancreatic cancer Duan et al. (2013); Shi and Cao, (2014); Wang et al. (2018); Yang et al. (2016); Yi et al. (2015).

TMEM170B, a transmembrane protein and a member of the TMEM170 family, is composed of 132 amino acids, with sequences that are highly conserved from invertebrates to mammals. Its important paralog is TMEM170A, which has been reported to be a new regulator of endoplasmic reticulum (ER) and nuclear envelope morphogenesis Christodoulou et al. (2016). A transcriptome-wide association study revealed that TMEM170A expression is significantly associated with the risk of pancreatic cancer Zhong et al. (2020). Moreover, a recent study suggested that low TMEM170B expression is an independent predictor of poor overall survival (OS) in patients with breast carcinoma (BRCA) (*p* < 0.01) and that TMEM170B could act as a tumor suppressor to antagonize the protumorigenic Wnt/β-catenin signaling pathway Li et al. (2018). Therefore, TMEM170B is a potentially attractive therapeutic target for future anticancer drugs although its role in pancreatic cancer remains unknown.

Hence, the present study aimed to investigate the role of TMEM170B as a prognostic biomarker and therapeutic target in pancreatic cancer and explore the possible underlying mechanisms of TMEM170B in the development of pancreatic cancer.

## 2 Material and Methods

### 2.1 Patients and Histological Specimens

After surgical resection at the Sichuan Provincial People’s Hospital between January 2018 and August 2020, 38 fresh paired tissue and adjacent nontumor tissue samples were obtained and divided into two groups for preservation in 10% formalin for histological examination and immediate freezing in liquid nitrogen and storage at –80°C until further use for qRT-PCR. All patients with pancreatic adenocarcinoma (PAAD) were histologically and clinically diagnosed and assessed according to the TNM staging system (7^th^ version) of the International Union against Cancer. Follow-up data for the patients were collected until 31 October 2021 or their date of death. Demographic, clinical, and blood parameters of all the patients were collected on the day of admission. Clinicopathological data for the patients with pancreatic ductal adenocarcinoma are presented in Table 1. OS was defined as the interval from the postoperative period until death or the last follow-up date. Disease-free survival (DFS) was calculated from post-operation until disease progression, death, or last follow-up.

**TABLE 1:** Association between TMEM170B expression and clinicopathological characteristics in 38 pancreatic adenocarcinoma patients.

Variable	All Patients (*n* = 38) (%)	TMEM170B expression		*p* Value (χ2-test)
		Positive (+) (*n* = 24)	Negative (−) (*n* = 14)	
Gender (male/female)	25 (65.8)/13 (34.2)	17 (70.8)/7 (29.2)	8 (57.1)/6 (42.9)	0.61
Age (years) (＜60/≥60)	18 (47.4)/20 (52.6)	11 (45.8)/13 (54.2)	7 (50)/7 (50)	0.8
Tumor size (cm) (＜5/≥5)	23 (60.5)/15 (39.5)	14 (58.3)/10 (41.7)	9 (64.3)/5 (35.7)	0.72
Tumor number (Single/Multiple)	27 (71.1)/11 (28.9)	18 (75)/5 (25)	9 (64.3)/6 (35.7)	0.40
Tumor cellularity (＜61%/≥61%)	23 (60.5)/15 (39.5)	17 (70.8)/7 (29.2)	6 (42.9)/8 (57.1)	0.09
Differentiation				
Well	7 (18.4)	5 (20.8)	2 (14.3)	
Moderate	22 (62.9)	17 (70.8)	5 (35.7)	0.014^*^
Poor	9 (23.7)	2 (8.4)	7 (50)	
pT classification				
T1–2	5 (13.2)	4 (16.7)	1 (7.1)	
T3	17 (44.7)	12 (50)	5 (35.7)	0.33
T4	16 (42.1)	8 (33.3)	8 (57.2)	0.94
Lymph node metastasis (Absent/Present)	22 (62.9)/16 (37.1)	14 (58.3)/10 (41.7)	8 (57.2)/6 (42.8)	
pTNM stage I-II/III-IV	11 (28.9)/27 (71.1)	8 (33.3)/16 (66.7)	3 (21.4)/11 (78.6)	0.68
CEA (＜5/≥5)	21 (55.3)/17 (44.7)	15 (62.5)/9 (37.5)	6 (42.8)/8 (57.2)	0.24
CA19-9 (＜35/≥35)	17 (44.7)/21 (55.3)	10 (41.7)/14 (58.3)	7 (50)/7 (50)	0.62
Cancer progression, n (%) (no/yes)	26 (68.4)/12 (31.6)	22 (91.7)/2 (8.3)	4 (28.6)/10 (71.4)	<0.001
Death, n (%) (no/yes)	29 (76.3)/9 (23.7)	23 (95.8)/1 (4.2)	6 (42.9)/8 (57.1)	<0.001

NOTE: p: primary; TNM: tumor-node-metastasis; CEA: carcinoembryonic antigen; CA19-9: carbohydrate antigen 19–9; *: *p* < 0.05.

### 2.2 Data Source and Processing

To assess TMEM170B mRNA levels in PAAD tissues and normal tissues, the online tools employed were provided by the Sangerbox (http://www.sangerbox.com/tool), based on The Cancer Genome Atlas (TCGA) (https://tcga-data.nci.nih.gov/) database, and by the Gene Expression database of Normal and Tumor tissues 2 (GENT2, http://gent2.appex.kr), based on the Gene Expression Omnibus (GEO) (http://www.ncbi.nlm.nih.gov/geo/) database. Gene presentation datasets (GSE32676, GSE16515, and GSE71729) were retrieved from the GEO database. Differentially expressed genes (DEGs) were screened with the adjusted *p*-value of<0.01 and |log fold change| of ≥2.

### 2.3 Cell Culture

Human pancreatic cancer cell lines PANC-1, AsPC-1, and BxPC-3 and the human pancreatic duct cell line HPDE6-C7 were obtained from the American Tissue Culture Collection (Manassas, VA, United States). All cells were cultured in Roswell Park Memorial Institute (RPMI)-1,640 medium containing 10% fetal bovine serum, followed by incubation at 37°C in 5% CO_2_. After a few passages (2–4), the cells in the logarithmic growth phase were used for further experiments.

### 2.4 RNA Extraction and Quantitative Real-Time Polymerase Chain Reaction (RT-PCR)

Total RNA from fresh frozen tissues and cells was extracted using TRIzol reagent (Invitrogen, NY, United States) according to the manufacturer’s instructions. RNA was quantified using a NanoDrop (Thermo Fisher Scientific, MA, United States). Subsequently, 1 μg of total RNA was used to produce cDNA using PrimeScript RT reagent (Takara, Kusatsu, and Japan), and qRT-PCR was performed using TB Green™ Premix Ex Taq™ II (Takara, Japan) in the CFX96 Real-time System (Bio-Rad). β-Actin was used for normalization purposes. Primers were designed based on the TMEM170B and β-Actin mRNA sequences in GenBank. The primers used were: TMEM170B, forward primer: 5′-TTC​CTC​TGG​GCT​CTC​TTC​TCT-3′, reverse primer: 5′-CTG​CTG​CAC​TGG​TAA​TCA​TCG-3′, β-Actin, forward primer: 5′-CCT​GAA​GTA​CCC​CAT​CGA​GC-3′, reverse primer: 5′-AGG​GAT​AGC​ACA​GCC​TGG​AT-3′. The relative expression levels of TMEM170B and β-actin were calculated using the comparative CT (2^−ΔΔCT^) method. Each sample was analyzed in triplicate.

### 2.5 Immunohistochemistry

Tissue samples were fixed in 10% formalin for at least 24 h, embedded in paraffin, and sections of 3-μm thickness were obtained. The tissue sections were then placed in Tris-ethylenediaminetetraacetic acid (pH 9.0) antigen repair solution and repaired in a pressure cooker for 5 min. Endogenous peroxidase activity was blocked with 3% H_2_O_2_ solution for 25 min at room temperature in a dark condition. Thereafter, the sections were incubated with 10% goat serum (HyClone, United States) in phosphate buffered saline (PBS) at room temperature for 1 h. The sections were then incubated overnight at 4°C with anti-TMEM170B antibody (1:100 dilution, PA5-63072, Thermo Fisher Scientific). After washing off the antibodies, the sections were incubated with rabbit secondary antibodies at 37°C.

The histology of the different tissues was analyzed microscopically (BX51, Olympus, Tokyo, Japan). Image capturing was performed using DP2-BSW software (Olympus). The immunohistochemistry (IHC) scores were obtained using a semi-quantitative method by comparing the staining intensity with the proportion of the stained cells. The H score system was employed for scoring the positive cell distribution as follows: the recording of the distribution (positive cell percentage) was determined using A = 0–100; the staining intensity was evaluated as B and scored as follows: strong (dark brown): 3; mild (brown): 2; light (light brown): 1; and none (not stained): 0. The following formula was used to determine the H scores in terms of intensity and distribution: H score = 1 × A1 +2 × A2 +3 × A3, 0 ≤ H ≤ 300.

### 2.6 Immunofluorescence

The tissue sections and cells on glass coverslips were fixed with 4% paraformaldehyde for 20 min and permeabilized with 0.1% Triton X-100 for 15 min at room temperature. After blocking with 5% BSA for 60 min at room temperature, the sections and cells were incubated overnight at 4°C with anti-TMEM170B (1:200 dilution), anti-CD4^+^(1:200 dilution, Proteintech)/anti-CD8^+^(1:200 dilution, Proteintech)/anti-CD11b (1:100 dilution, Proteintech)/anti-CD33 (1:25 dilution, Proteintech). After three washes with PBS, the sections and cells were stained with fluorescent labeled secondary antibodies for 1 h at 37°C. The coverslips were stained with 4′,6-diamidino-2-phenylindole (Sigma) and examined under a confocal microscope (LSM 800, Zeiss) with a 63 × /1.40 oil-immersion objective lens.

### 2.7 Correlation and Gene Enrichment Analysis

Correlation analysis between TMEM170B and other mRNAs in PAAD was performed using TCGA data, and the Pearson correlation coefficient was calculated. The top 300 genes that were most positively associated with TMEM170B were selected for enrichment analysis to reflect the role of TMEM170B. Gene Ontology (GO) analysis and Kyoto Encyclopedia of Genes and Genomes (KEGG) analysis of genes were performed using the functional annotation tool in the Database for Annotation, Visualization, and Integrated Discovery (DAVID, https://david.ncifcrf.gov) and Gene Set Enrichment Analysis (GSEA) Reimand et al. (2019). The Reactome Knowledgebase provides both as an archive of biological processes and as a tool for discovering functional relationships in data Jassal et al. (2020). GSEA was performed using the gseGO, gseKEGG, and gsePathway functions of the R package “clusterProfiler.”

### 2.8 Immune Cells Infiltration Analysis

The level of immune and stromal fraction was scored by Estimation of Stromal Immune cells in MAlignant Tumor tissues using Expression data (ESTIMATE) based on log2-transformed TPM data. Tumor IMmune Estimation Resource 2.0 (TIMER 2.0, http://timer.cistrome.org/) algorithm database provided immune cell infiltration expression profiles by TIMER, CIBERSORT Chen et al. (2018), Microenvironment Cell Populations-counter (MCP-counter) Becht et al. (2016), EPIC Racle et al. (2017), these algorithms were used to determine the Spearman correlation between TMEM170B expression levels in PAAD and the infiltration of different immune cells. The relationship between immune cell infiltration and prognosis of patients was further analyzed. *p* values <0.05 were considered to denote statistical significance.

### 2.9 Statistical Analysis

The clinical characteristics of the patients were compared using the Fisher’s exact test for categorical variables and Wilcoxon rank-sum test for continuous variables. The continuous and categorical variables were expressed as mean (standard deviation) and proportions (percentages), respectively. Multivariate time-dependent Cox proportional DFS and OS hazard ratios (HRs) were fitted based on significant univariate factors. Survival analyses were performed using the Kaplan-Meier method and log rank test. The IHC results were quantitatively analyzed using ImageJ software (MEDIA CYBERNETICS, United States). All *p* values were two-sided tests, with *p* < 0.05 indicating statistical significance. All data analyses were performed using SPSS version 27.0 (IBM Corp., NY, United States) and R 3.6.0 (The R Foundation for Statistical Computing, Vienna, Austria).

## 3 Results

### 3.1 Downregulation of TMEM170B Expression in Human Pancreatic Cancer

**FIGURE 1:**
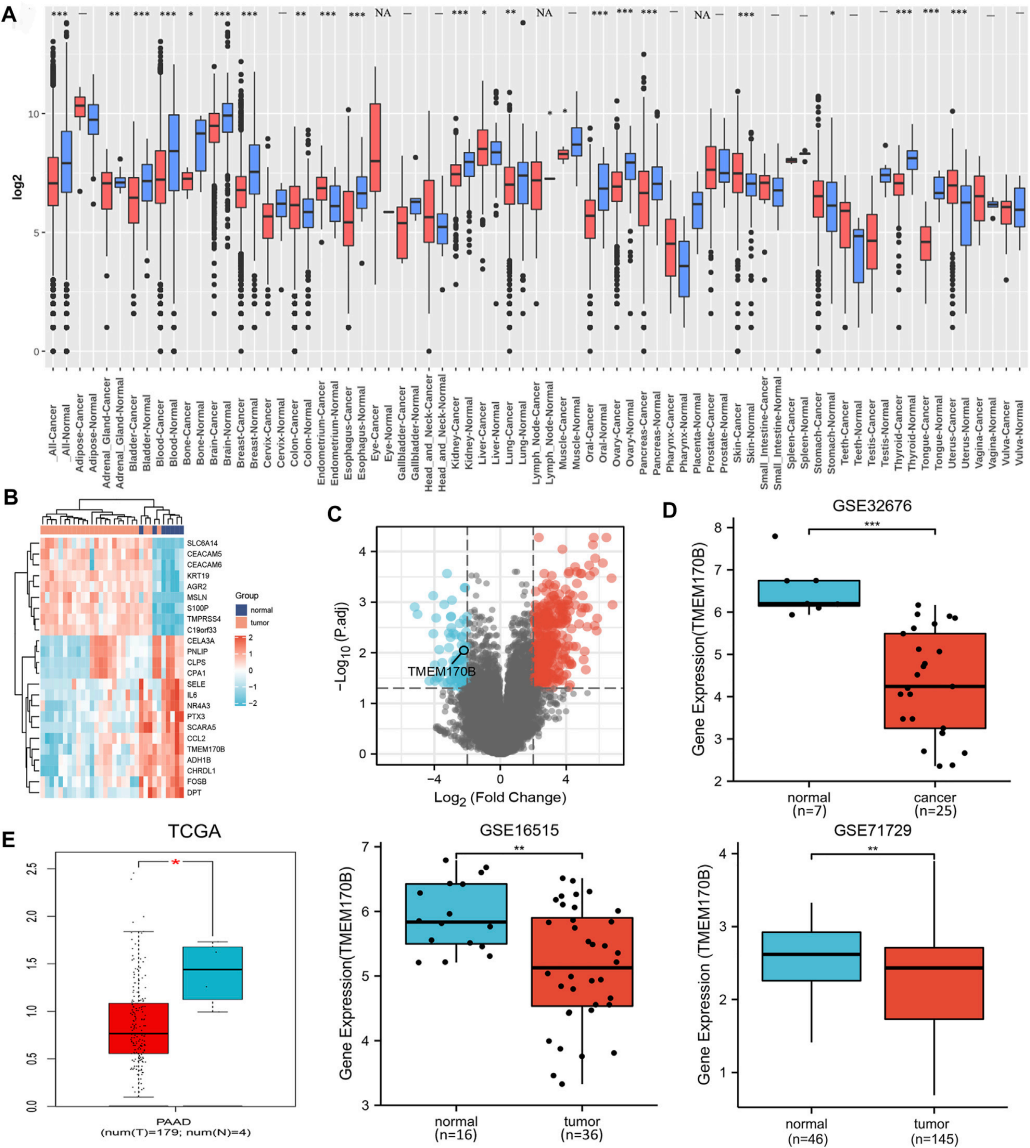
TMEM170B mRNA expression levels in different tumors and corresponding adjacent normal tissues. **(A)** The pan-cancer TMEM170B expression status was analyzed using GENT2 based on the GEO database. **(B)** The heat map of the top 24 ranked DEGs. **(C)** The volcano plot of all DEGs, red represents up-regulated, blue represents down-regulated genes. **(D,E)** The expressions of TMEM170B in pancreatic adenocarcinoma and the corresponding nontumor tissues were inferred by analyzing the TCGA and GEO databases. ns: not significant. *p < 0.05; **p < 0.01; ***p < 0.001.

GENT2 tool was utilized to examine the mRNA levels of TMEM170B in pan-cancer tissues. We first determined that TMEM170B was significantly downregulated in conditions such as PAAD, breast cancer, oral cancer, ovary cancer, and thyrioid cancer (Figure 1A). In pancreatic cancer data set GSE32676 from the GEO, the top 24 ranked DEGs were selected to form a heat map (Figure 1B). All DEGs constituted the volcano plot of the differential genes (Figure 1C). Compared with normal tissues, TMEM170B expression was frequently downregulated in the PAAD tissues from GSE32676 (*p* < 0.001) (Figure 1D). In addition, consistent with the above results, TMEM170B was obviously low expressed in PAAD tissues from TCGA (*p* = 0.023), GSE16515 (*p* = 0.005), and GSE32676 (*p* < 0.001) datasets (Figure 1E).

**FIGURE 2:**
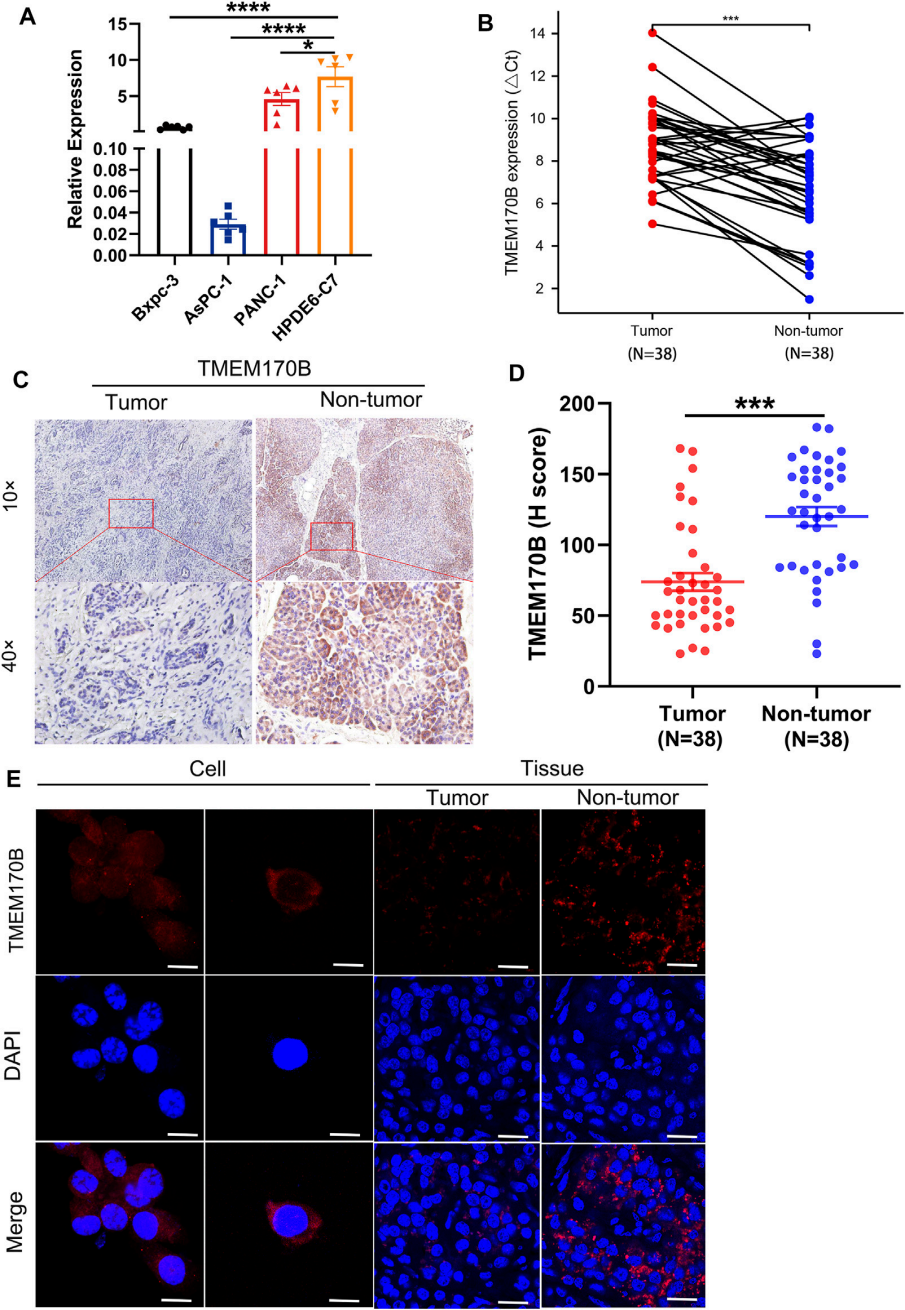
TMEM170B expression was downregulated in human pancreatic cancer cell lines and tissues. **(A)** TMEM170B mRNA levels were low in pancreatic cancer cell lines. **(B)** Real-time PCR analysis of TMEM170B mRNA expression in 38 pairs of pancreatic adenocarcinoma (PAAD) tissue and paired nontumor tissue. **(C)** Representative immunohistochemistry (IHC) images of TMEM170B in 38 pairs of matched tumor tissues and adjacent nontumor tissues. **(D)** IHC quantification was performed using ImageJ software, and TMEM170B protein expression in PAAD tissues was lower than that in nontumor tissues. **(E)** The subcellular localization of TMEM170B was analyzed with immunostaining (cell: scale bar, 20 μm; tissue: scale bar, 30 μm); ns: not significant. *p < 0.05; **p < 0.01; ***p < 0.001.

To determine the low expression of TMEM170B in human pancreatic cancer cell lines and tissues, the mRNA and protein levels of TMEM170B were assayed using RT-PCR and IHC, respectively. Our results revealed that TMEM170B expression was considerably lower in pancreatic cancer cell lines and tissues than in normal pancreatic duct epithelial cells and normal pancreatic tissues, respectively (Figures 2A–E). Moreover, IHC and IF demonstrated that TMEM170B was localized in the plasma membrane and cytoplasmic regions of pancreatic cancer cells (Figures 2C,E) and that 63.2% of the pancreatic cancer tissue samples were TMEM170B positive (Figure 2D).

The above results confirmed that TMEM170B is expressed at lower levels in PAAD when compared with its levels in adjacent normal tissues.

### 3.2 Significance of TMEM170B Expression and Prognosis of PAAD in the TCGA Cohort

**FIGURE 3:**
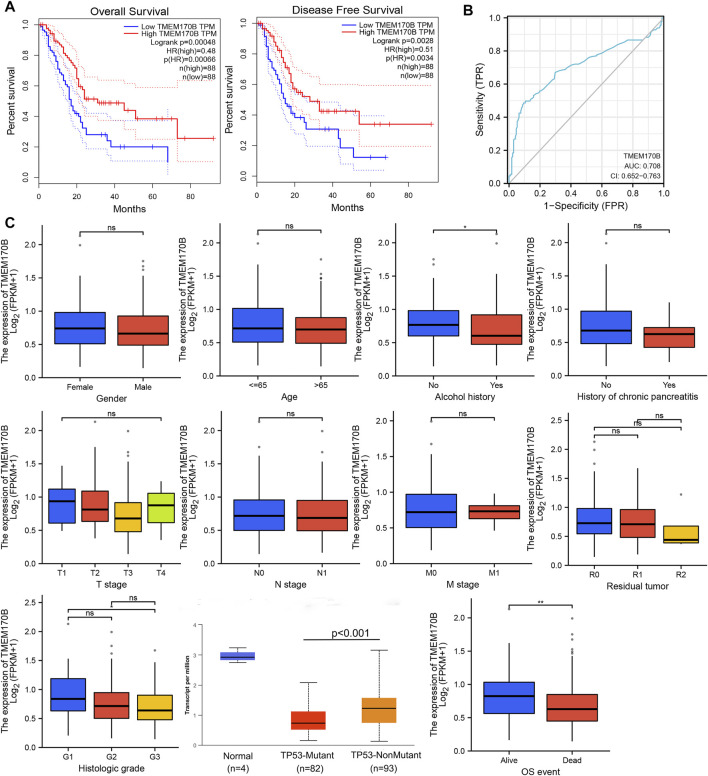
Significance of TMEM170B expression and prognosis of pancreatic adenocarcinoma (PAAD) in the TCGA cohort. **(A)** The Kaplan–Meier survival curve analysis showed that low TMEM170B expression was related to poor overall survival and relapse-free survival. **(B)** Receiver operator characteristic curve analysis of TMEM170B expression in PAAD. **(C)** Relationship between TMEM170B expression and clinicopathological factors. ns: not significant; *p < 0.05; **p < 0.01.

The TCGA cohort was used to further investigate TMEM170B expression and its correlation with clinical prognosis among patients with PAAD. Patients with low TMEM170B expression had worse OS (*p* < 0.001) and disease-free survival (DFS) (*p* = 0.0028) than those with high TMEM170B expression (Figure 3A). The receiver operating characteristic (ROC) analysis showed that TMEM170B expression had robust prognostic predictive performance (areas under the ROC curve (AUC) = 0.708, 95% CI = 0.652–0.763, Figure 3B). Subsequently, the clinical significance of TMEM170B expression in patients with PAAD was systematically assessed using this cohort. The downregulation of TMEM170B expression was common in human PAAD. Low TMEM170B expression group usually have a history of alcohol abuse (*p* < 0.05), higher histologic grade (*p* < 0.05, G3 vs. G1) and more dead event (*p* < 0.001), but TMEM170B expression is not associated with sex, age, primary tumor (pT) stage, lymph node invasion, metastasis, and residual tumor (Figure 3C).

### 3.3 Association Between TMEM170B Expression and Clinicopathological Features of PAAD

**FIGURE 4:**
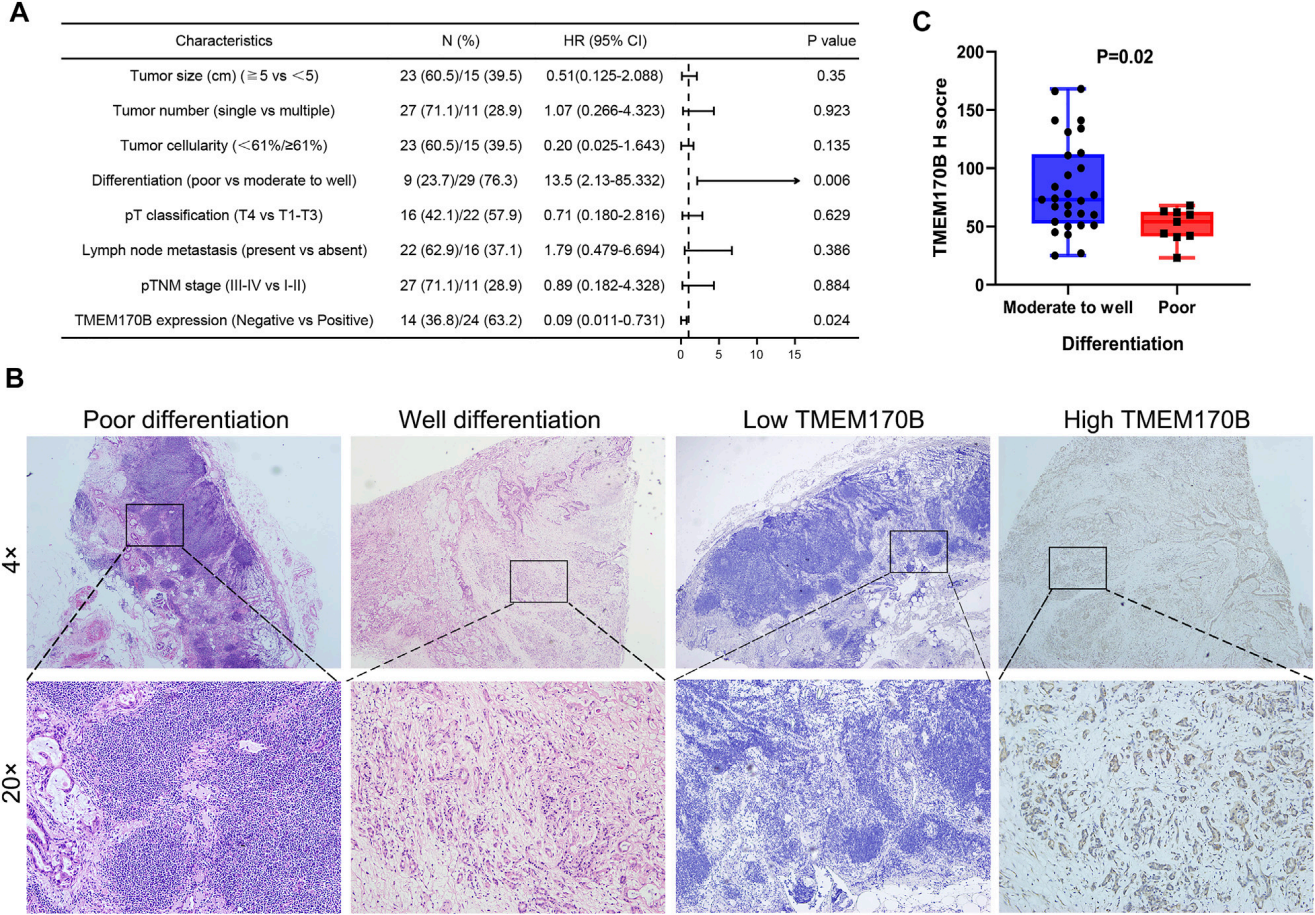
Correlation of TMEM170B expression with the degree of differentiation in PAAD. **(A)** The Cox regression analysis of the forecast factors of OS by univariate analysis. **(B)** The histopathological comparison of the degrees of differentiation and TMEM170B expression of the same specimen. **(C)** TMEM170B Expression in Moderate to well differentiation group and poor group.

Tumor tissues were divided into low and high TMEM170B expression based on the median values of H-score of each core. Since the median of H-score was 61, the patients were divided into two groups: H scores <61 was defined as low TMEM170B group, H scores ≥61 was high TMEM170B group, respectively. A total of 38 patients with complete clinical data were included to analyze the relationship between TMEM170B expression and various clinicopathological factors using the chi-squared test. The results illustrated that low TMEM170B expression had a significant correlation with poor differentiation (*p* = 0.014). Nevertheless, TMEM170B expression was not significantly associated with sex, age, tumor size or number, pathological tumor-node-metastasis (pTNM) stage, pT classification, serum carcinoembryonic antigen, and carbohydrate antigen 19–9 (CA19-9) levels (Table 1). The Cox regression analysis of the forecast factors of OS by univariate analysis was showed in Figure 4A. A histopathological comparison of the degrees of differentiation and TMEM170B expression of tumor tissues also indicates that low expression of TMEM170B in PAAD was correlated with less tumor differentiation (Figure 4B). Moderate to well differentiation groups had generally higher TMEM170B expression relative to poor groups (*p* = 0.02, Figure 4C).

### 3.4 Correlation of TMEM170B Expression With Clinical Prognosis in PAAD

**FIGURE 5:**
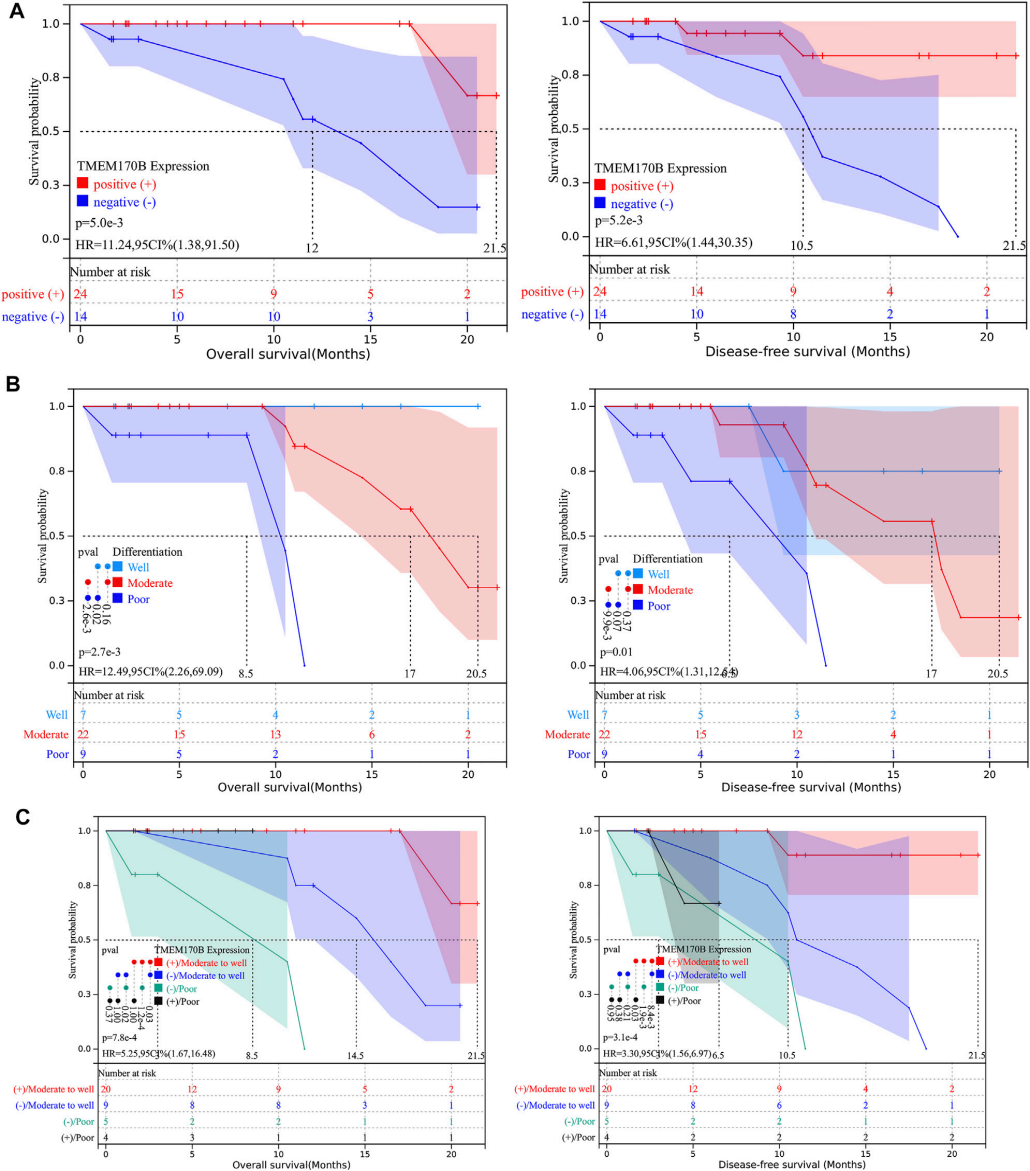
TMEM170B expression and the degree of differentiation correlated with survival outcomes in our cohort. **(A)** Low TMEM170B expression was significantly associated with poor clinical outcomes. **(B)** The outcomes in the poor differentiation group were worse than those of the moderate and high differentiation groups. **(C)** Association of TMEM170B expression with the outcomes in poor differentiation or moderate to high differentiation: Subgroup analysis.

To determine whether TMEM170B expression levels were associated with clinical PAAD progression, we checked the association between TMEM170B expression and the clinical outcomes in our patients. Kaplan-Meier analysis revealed that patients in the TMEM170B-negative group had significantly worse OS (*p* = 0.005) and DFS (*p* = 0.0052) than those in the TMEM170B-positive group (Figure 5A). Univariate analysis identified poor differentiation (*p* = 0.006) and low TMEM170B expression (*p* = 0.024) as factors significantly associated with OS. Multivariate analysis indicated that differentiation [HR = 7.26, 95% CI = 1.141–46.147; *p* = 0.036] and TMEM170B expression (HR = 0.12, 95% CI = 0.014–0.995, and *p* = 0.049) were independent prognostic factors for OS. Similarly, univariate analysis demonstrated that poor differentiation (*p* = 0.013) and low TMEM170B expression (*p* = 0.016) were pivotal factors for DFS. However, multivariate analysis signified that only TMEM170B expression was an independent predictor (HR 0.19, 95% CI 0.04–0.91; *p* = 0.038) and not differentiation (HR 3.29, 95% CI 0.848–12.734; *p* = 0.085) (Table 2). Kaplan-Meier analysis also showed that moderate and poor differentiation had worse prognostic performance when compared with well differentiation (Figure 5B). In subgroup analysis, the moderate to well differentiation group with high TMEM170B expression achieved an average OS of 9.5 months, which was longer than that of the other groups (Figure 5C).

**TABLE 2:** Univariate and multivariate Cox analyses of clinicopathological factors and clinical outcomes.

OS characteristics	Univariate analysis			Multivariate analysis		
	HR	95% CI	*p*-value	HR	95% CI	*p*-value
Gender (male vs. female)	1.35	0.319–5.728	0.682			
Age (years) (≧60 vs .< 60)	0.65	0.173–2.427	0.337			
			
Tumor size (cm) (≧5 vs. ＜5)	0.51	0.125–2.088	0.35			
Tumor number (single vs. multiple)	1.07	0.266–4.323	0.923			
Tumor cellularity (＜61% vs .≥ 61%)	0.20	0.025–1.643	0.135			
Differentiation (poor vs. moderate to well)	13.5	2.13–85.332	0.006^**^	7.26	1.141–46.147	0.036^*^
pT classification (T4 vs. T1-T3)	0.71	0.180–2.816	0.629			
Lymph node metastasis (present vs. absent)	1.79	0.479–6.694	0.386			
pTNM stage (III-IV vs. I-II)	0.89	0.182–4.328	0.884			
CEA (≧5 vs. < 5)	0.45	0.114–1.735	0.244			
CA19-9 (≧35 vs. < 35)	1.11	0.295–4.17	0.877			
TMEM170B expression (Negative vs. Positive)	0.09	0.011–0.731	0.024^*^	0.12	0.014–0.995	0.049^*^
**DFS**	**Univariate Analysis**			**Multivariate Analysis**		
		**HR**	**95% CI**	***p*** **-value**	**HR**	**95% CI**	***p*** **-value**
Gender (male vs. female)	0.6	0.16–2.217	0.44			
Age (years) (≧60 vs .< 60)	0.51	0.160–1.640	0.26			
Tumor size (cm) (≧5 vs. ＜5)	0.78	0.237–2.546	0.677			
Tumor number (single vs. multiple)	1.14	0.358–3.605	0.828			
Tumor cellularity (＜61% vs .≥ 61%)	0.137	0.018–1.064	0.057			
Differentiation (poor vs. moderate to well)	5.43	1.421–20.776	0.013^*^	3.29	0.848–12.734	0.085
pT classification (T4 vs. T1-T3	0.78	0.216–2.812	0.704			
Lymph node metastasis (present vs. absent)	1.66	0.524–5.243	0.39			
pTNM stage (III-IV vs. I-II)	0.81	0.169–3.868	0.791			
CEA (≧5 vs. < 5)	0.47	0.124–1.742	0.256			
CA19-9 (≧35 vs. < 35)	2.45	0.716–8.398	0.153			
TMEM170B expression (Negative vs. Positive)	0.15	0.033–0.702	0.016^*^	0.19	0.04–0.910	0.038^*^

Notes: OS, overall survival; DFS, Disease-free survival; HR, hazard ratio; CI, confidence interval, *p < 0.05, **p < 0.01.

### 3.5 Correlation and Enrichment Analyses of TMEM170B in PAAD

**FIGURE 6:**
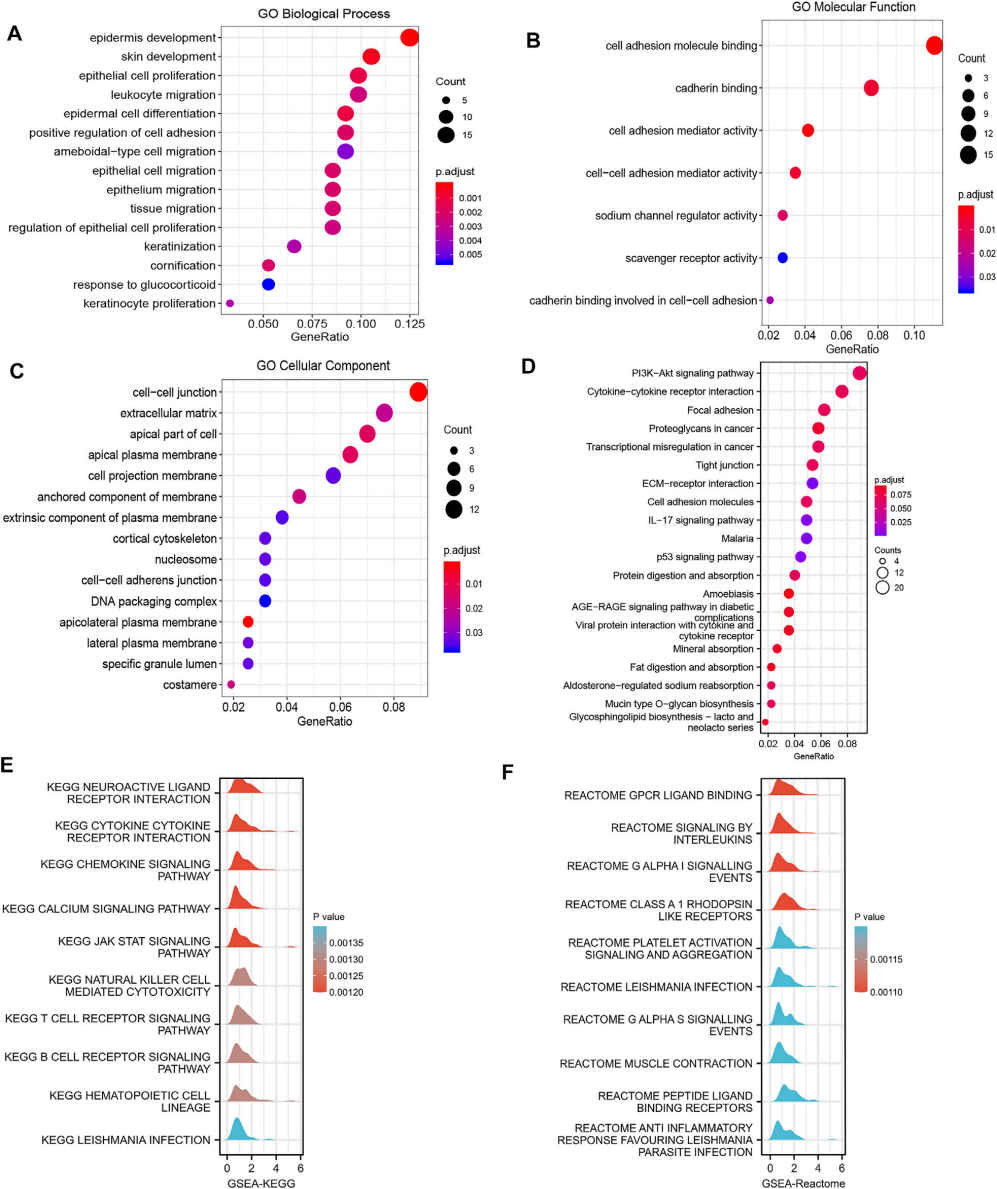
Function and pathway enrichment analyses of TMEM170B in pancreatic adenocarcinoma. **(A–C)** Significant GO terms of the top 300 genes most positively associated with TMEM170B, including biological processes, molecular function, and cell component. **(D)** Significant KEGG pathways of TMEM170B-related genes. **(E,F)** Significant GSEA results of the top 300 genes most positively associated with TMEM170B, including KEGG pathways and Reactome pathways.

For functional and pathway enrichment analyses of TMEM170B-related molecules, GO terms and KEGG enrichment pathway were visualized using the cluster Profiler R software package. According to functional enrichment and GO analyses, the genes were mainly enriched during biological activities such as leukocyte migration, cell–cell adhesion mediator activity, ameboidal-type cell migration and epithelial cell migration, all of which were correlation with the activities of immune cells (Figures 6A–C). Moreover, the KEGG pathway mainly involved cytokine–cytokine receptor interaction, malaria, the IL-17 signaling pathway, and the p53 signaling pathway (Figure 6D). Most importantly, GSEA was used to further search for KEGG and Reactome pathways. GSEA-KEGG revealed that cytokine–cytokine receptor interaction, the chemokine signaling pathway, natural killer cell mediated cytotoxicity and the T cell receptor signaling pathway were significantly enriched (Figure 6E). Furthermore, G protein-coupled receptor (GPCR) ligand binding, interleukin signaling, and platelet activation signaling and aggregation were found to be significantly enriched in Reactome pathway analysis (Figure 6F). According the above results, we thus reasoned that TMEM170B may be involved in immune cell infiltration.

### 3.6 Correlation Between Immune Cell Infiltration, Immunotherapeutic Benefits and TMEM170B in PAAD

**FIGURE 7:**
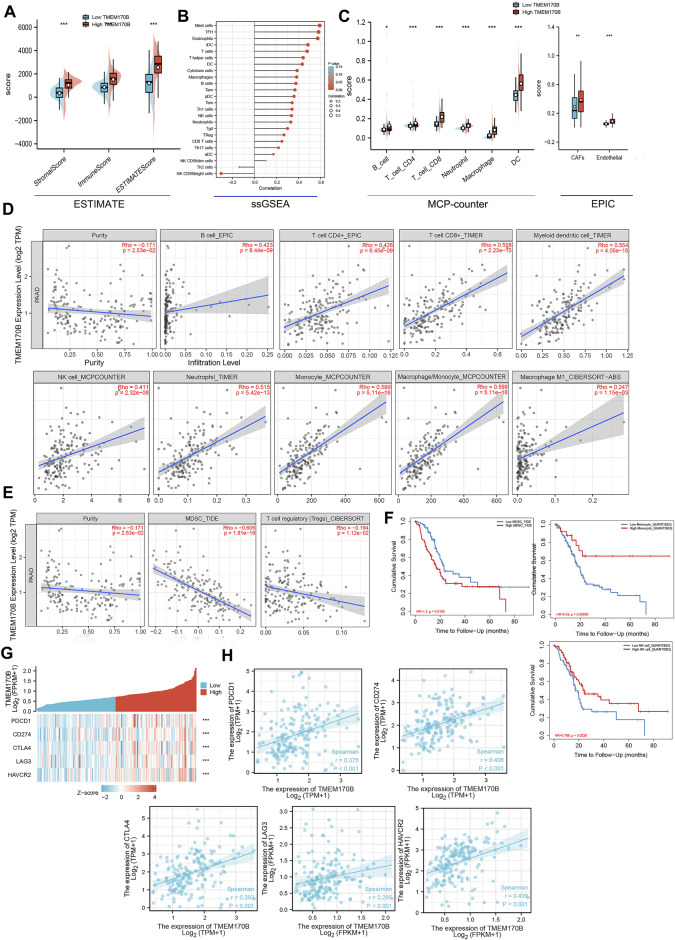
Correlation analysis between TMEM170B expression and immune infiltration of multiple immune cells. **(A)** Violin plot showing the differential StromalScore, ImmuneScore, and ESTIMATEScore of high and low TMEM170B expression. **(B)** The correlation between TMEM170B expression level and 24 immune cell types. **(C–D)** The correlation between TMEM170B expression and antitumor immune cell infiltration, including that of B cells, CD8^+^T cells, CD4^+^T cells, myeloid dendritic cells, natural killer cells, neutrophils, monocyte and M1 macrophages. **(E)** The correlation between TMEM170B expression and the infiltration of immunosuppressive cells, including myeloid-derived suppressor cells and regulatory T cells. **(F)** Kaplan-Meier analysis results based on the extent of monocyte, natural killer cells and yeloid-derived suppressor cells infiltration in PAAD. Red represents high immune cells infiltration, and blue represents low immune cells infiltration. **(G,H)** The gene-by-gene correlation analysis of common ICP genes and TMEM170B expression. *p < 0.05; **p < 0.01; ***p < 0.001.

Immune cells in the tumor microenvironment play a central role in tumor-associated immune responses, which may regulate tumor progression and determine the prognosis. We further explore the correlation between TMEM170B expression and the infiltration level of different immune cells in PAAD. First and foremost, immuneScore, StromalScore and ESTIMATEScore were in the TMEM170B high-expression cluster than in the TMEM170B low-expression cluster (Figure 7A). ssGSEA with Spearman’s rank correlation was applied to the analysis of correlation between TMEM170B expression and infiltration levels of 24 immune cell types, the result showed that TMEM170B expression was negatively correlated with the infiltration of Th2 cells and NK CD56bright cells (Figure 7B). By TIMER, CIBERSORT, MCP-counter and EPIC algorithms, the results indicated that TMEM170B expression was positively correlated with the infiltration of antitumor immune cells, including B cells, CD8^+^T cells, CD4^+^T cells, dendritic cells (DCs), natural killer cells (NKs), neutrophils, monocyte, and M1 macrophage (Figures 7C,D). Moreover, a negative correlation between TMEM170B expression and immune infiltration of myeloid-derived suppressor cells (MDSCs) and T cell regulatory (Tregs) was noted (Figure 7E). Elevated monocyte and NKs and reduced MDSCs infiltration were associated with good prognosis in patients with PAAD (Figure 7F). The expression of immune checkpoint (ICP) genes, such as PDCD1 (PD1), CD274 (PDL1), CTLA4, LAG3, and HAVCR2 (TIM3) has been utilized in predicting the response of patients to immune checkpoints therapy in a variety of cancers including PAAD. In gene-by-gene correlation analysis, these common ICP genes were positively correlated with TMEM170B expression (Figures 7G,H). This illustrated that TMEM170B might have value in predicting immunotherapeutic benefits.

### 3.7 TMEM170B Expression Is Correlated With the Infiltration Levels of CD4^+^ T Cells, CD8^+^T Cells, and MDSCs

**FIGURE 8:**
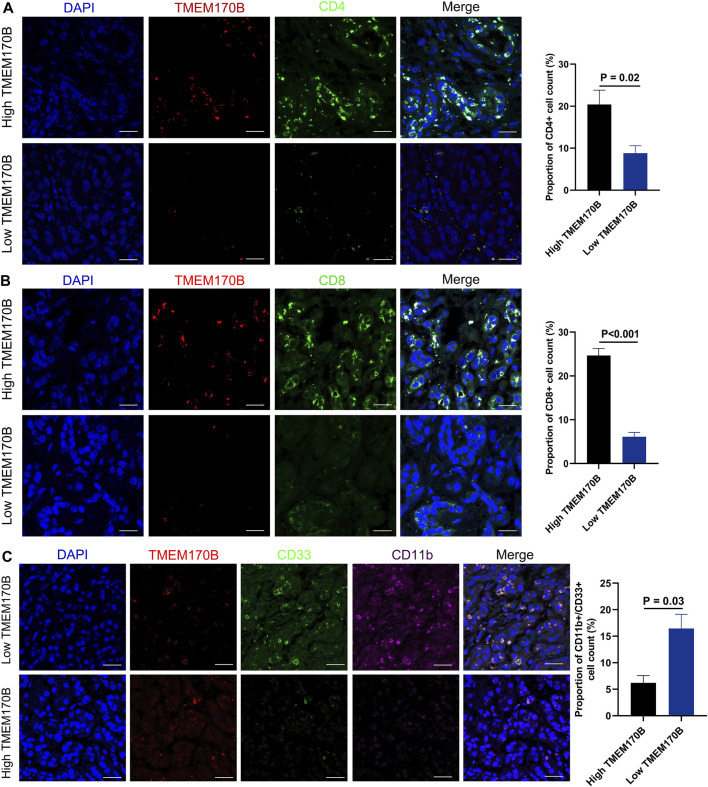
Correlation Between TMEM170B Expression and the Infiltration Levels of CD4^+^ T Cells, CD8+T cells, and MDSCs in PAAD tissues. **(A)** TMEM170B expression was positively correlated with the infiltration of CD4^+^T cells. **(B)** TMEM170B expression was positively correlated with the infiltration of CD8^+^T cells. **(C)** TMEM170B expression was negatively correlated with the infiltration of MDSCs. Scale bar, 30 μm.

Next, the immunofluorescence method was performed to validate whether immune cell infiltration was correlated with TMEM170B expression in our cohort. Since T cells and MDSCs were the most prevalent cells among the infiltrated immune cells correlated with TMEM170B expression, and previous studies have shown that CD4^+^T Cells, CD8^+^T cells and MDSCs are the major factors responsible for impacting pancreatic cancer progression, invasiveness, metastasis, and patients^’^ survival Foley et al. (2016; Thyagarajan et al. (2019), we further explored the correlation between CD4^+^T Cells, CD8^+^T cells, and MDSCs infiltration and TMEM170B expression. It was observed in tumor center that the tumor tissue presented a high TMEM170B expression level, the infiltration Levels of CD4^+^T Cells, CD8^+^T cells was relatively active. While infiltration of CD4^+^T Cells, CD8^+^T cells was significantly low in areas with a corresponding low TMEM170B expression status (Figures 8A,B). Conversely, we observed that the MDSCs (labeled by CD11b^+^CD33^+^) was obviously recruited in tumor regions with low TMEM170B expression level (Figure 8C). Therefore, these results preliminarily verified our inferences in the above analysis that TMEM170B was indeed related to CD4^+^, CD8^+^T cells recruitment, and MDSCs blocking.

## 4 Discussion

Owing to the surge in the prevalence of obesity, diabetes, alcohol consumption, smoking, and pancreatitis, both the incidence and mortality rates of pancreatic cancer have increased steadily in Europe and the United States over the past decades Connor and Gallinger, (2015). Despite the recent therapeutic advances, the prognosis of the patients is yet to improve significantly. PAAD is a highly invasive and metastatic type of pancreatic cancer that results in poor clinical outcomes in the patients Hackeng et al. (2016). However, its pathogenesis and underlying mechanisms are yet to be elucidated Fang et al. (2017; Hoskins et al. (2016; Klein et al. (2018; Zheng et al. (2016). Thus, there is an urgent need to identify novel markers and therapeutic targets for the early diagnosis of pancreatic cancer and prevention of metastasis. Furthermore, research in this direction is likely to aid in developing effective targeted molecular therapy for pancreatic cancer.

TMEM170B was mapped to human chromosome 6p24.2, which belongs to the TMEM family. TMEM170B and its paralog, TMEM170A, have shown diverse but specific expression profiles in different tumor cells and tissues and are involved in several biological processes, such as gene expression, invasion, proliferation, tumor apoptosis, normal cellular function, and disease pathogenesis Zhong et al. (2020). Current research has shown that TMEM170B negatively regulates canonical Wnt signaling in breast cancer cells and exerts an inhibitory effect on breast cancer growth by inhibiting CTNNB1 stabilization and nucleus translocation, which reduces the activity of Wnt targets Li et al. (2018).

The present study is the first to show that TMEM170B expression is significantly decreased in human pancreatic cancer cell lines and PAAD tissues. More importantly, univariate and multivariate analyses identified TMEM170B expression level and the degree of differentiation as the independent prognostic factors for OS and DFS in patients with PAAD. This finding suggests that low TMEM170B expression and poor differentiation are significantly related to poor prognosis. Consistently, low expression levels of TMEM170B are closely associated with poor differentiation and *TP53* mutation in patients with PAAD.

As a tumor suppressor gene, the tumor suppressor effect of *TP53* gene has been demonstrated in multiple tumors Olivier et al. (2010). Mutations in *TP53* indicate a poor prognosis in pancreatic cancer Guerra et al. (2011; Lu et al. (2020). Our study showed that low expression of TMEM170B is significantly related to *TP53* mutations. KEGG analysis also revealed that TMEM170B is involved in the p53 signaling pathway, which indicates that the presence of TMEM170B may inhibit the occurrence of *TP53* mutations and then exert a tumor suppressor effect in PAAD.

The tumor microenvironment (TME) and its individual immune cells play important roles in tumor initiation, progression, and metastasis Pattabiraman and Weinberg, (2014). A study showed that immune suppressive cells in pancreatic cancer maintain a tumor-friendly environment, with the majority of immune cells being macrophages and exhausted lymphocytes, based on single-cell RNA sequencing Lin et al. (2020). As such, we explored the biological functions and complex mechanisms of TMEM170B, and the correlation between immune cell infiltration and TMEM170B. GO results showed that TMEM170B is involved in leukocyte migration and cell–cell junction, whereas KEGG and GSEA indicated that TMEM170B is closely associated with immune-related pathways, cytokine–cytokine receptor interaction, chemokine signaling pathway, and T and B cell receptor signaling pathways in PAAD.

By analyzing the relationship between TMEM170B and immune cell infiltration, we found that low TMEM170B expression is often associated with MDSCs and Tregs infiltration into tumors, which aids in immune evasion. Moreover, low TMEM170B expression is linked to decreased levels of antitumor immune cells, such as B cells, CD8^+^T cells, CD4^+^T cells, NKs, DCs, and M1 monocytes. Therefore, activating TMEM170B may increase lymphocyte infiltration and accordingly decrease the immune suppressive cells within the TME. Tissue staining showed that TMEM170B had strong positive correlations with tumour-infiltrating CD4^+^ and CD8^+^ cells, and negative correlations with MDSCs.

Immunotherapy, which is used in cancer treatment to block the immune checkpoints such as programmed cell death-1 (PD-1) and programmed cell death-ligand 1(PD-L1), has shown persisting clinical responses and prolonging survival Feng et al. (2017). Hence, TMEM170B can be a potential prognostic biomarker and immunotherapy agent in combination therapy regimens to improve pancreatic cancer treatment.

In conclusion, this study is the first to confirm that human pancreatic cancer cells and tissues have decreased expressed of TMEM170B, which suggests that TMEM170B can serve as an independent prognostic predictor following surgery in patients with PAAD. Moreover, our results showed that the antitumor effects might be explained by the reduced number of immunosuppressive cells (MDSCs and Tregs) and the infiltration of antitumor immune cells (CD8^+^T cells, CD4^+^T cells, and M1 macrophage) into the TME. Thus, TMEM170B could be regarded as a novel target to address cancer progression. Future research endeavors to design novel drugs that can activate TMEM170B are expected to counteract the immunosuppressive microenvironment and improve the response to immunotherapy.

## Data Availability

The datasets presented in this study can be found in online repositories. The names of the repository/repositories and accession number(s) can be found in the article/Supplementary Material.

## References

[B1] Becht E. Giraldo N. A. Lacroix L. Buttard B. Elarouci N. Petitprez F. (2016). Estimating the Population Abundance of Tissue-Infiltrating Immune and Stromal Cell Populations Using Gene Expression. Genome Biol. 17, 218. 10.1186/s13059-016-1070-5 27765066 PMC5073889

[B2] Cassetta L. Kitamura T. (2018). Targeting Tumor-Associated Macrophages as a Potential Strategy to Enhance the Response to Immune Checkpoint Inhibitors. Front. Cel Dev. Biol. 6, 38. 10.3389/fcell.2018.00038

[B3] Chen B. Khodadoust M. S. Liu C. L. Newman A. M. Alizadeh A. A. (2018). Profiling Tumor Infiltrating Immune Cells with CIBERSORT. Methods Mol. Biol. 1711, 243–259. 10.1007/978-1-4939-7493-1_12 29344893 PMC5895181

[B4] Christodoulou A. Santarella-Mellwig R. Santama N. Mattaj I. W. (2016). Transmembrane Protein TMEM170A Is a Novel Regulator of ER and NE Morphogenesis in Human Cells. J. Cel Sci 129, 1552–1565. 10.1242/jcs.175273

[B5] Connor A. A. Gallinger S. (2015). Hereditary Pancreatic Cancer Syndromes. Surg. Oncol. Clin. North America 24, 733–764. 10.1016/j.soc.2015.06.007

[B6] Connor A. A. Gallinger S. (2021). Pancreatic Cancer Evolution and Heterogeneity: Integrating Omics and Clinical Data. Nat. Rev. Cancer 22, 131–142. 10.1038/s41568-021-00418-1 34789870

[B7] Duan L. Hu X.-q. Feng D.-y. Lei S.-y. Hu G.-h. (2013). GPC-1 May Serve as a Predictor of Perineural Invasion and a Prognosticator of Survival in Pancreatic Cancer. Asian J. Surg. 36, 7–12. 10.1016/j.asjsur.2012.08.001 23270819

[B8] Fang J. Jia J. Jia J. Makowski M. Xu M. Wang Z. (2017). Functional Characterization of a Multi-Cancer Risk Locus on chr5p15.33 Reveals Regulation of TERT by ZNF148. Nat. Commun. 8. 10.1038/ncomms15034

[B9] Feng M. Xiong G. Cao Z. Yang G. Zheng S. Song X. (2017). PD-1/PD-L1 and Immunotherapy for Pancreatic Cancer. Cancer Lett. 407, 57–65. 10.1016/j.canlet.2017.08.006 28826722

[B10] Foley K. Kim V. Jaffee E. Zheng L. (2016). Current Progress in Immunotherapy for Pancreatic Cancer. Cancer Lett. 381, 244–251. 10.1016/j.canlet.2015.12.020 26723878 PMC4919239

[B11] Guerra C. Collado M. Navas C. Schuhmacher A. J. Hernández-Porras I. Cañamero M. (2011). Pancreatitis-induced Inflammation Contributes to Pancreatic Cancer by Inhibiting Oncogene-Induced Senescence. Cancer Cell 19, 728–739. 10.1016/j.ccr.2011.05.011 21665147 PMC4890723

[B12] Hackeng W. M. Hruban R. H. Offerhaus G. J. A. Brosens L. A. A. (2016). Surgical and Molecular Pathology of Pancreatic Neoplasms. Diagn. Pathol. 11, 47. 10.1186/s13000-016-0497-z 27267993 PMC4897815

[B13] Hoskins J. W. Ibrahim A. Emmanuel M. A. Manmiller S. M. Wu Y. O’Neill M. (2016). Functional Characterization of a chr13q22.1 Pancreatic Cancer Risk Locus Reveals Long-Range Interaction and Allele-specific Effects onDIS3expression. Hum. Mol. Genet. 25, ddw300–4738. 10.1093/hmg/ddw300

[B14] Jassal B. Matthews L. Viteri G. Gong C. Lorente P. Fabregat A. (2020). The Reactome Pathway Knowledgebase. Nucleic Acids Res. 48, D498–D503. 10.1093/nar/gkz1031 31691815 PMC7145712

[B15] Klein A. P. Wolpin B. M. Risch H. A. Stolzenberg-Solomon R. Z. Mocci E. Zhang M. (2018). Genome-wide Meta-Analysis Identifies Five New Susceptibility Loci for Pancreatic Cancer. Nat. Commun. 9, 556. 10.1038/s41467-018-02942-5 29422604 PMC5805680

[B16] Li M. Han Y. Zhou H. Li X. Lin C. Zhang E. (2018). Transmembrane Protein 170B Is a Novel Breast Tumorigenesis Suppressor Gene that Inhibits the Wnt/β-Catenin Pathway. Cell Death Dis 9, 91. 10.1038/s41419-017-0128-y 29367600 PMC5833782

[B17] Lin W. Noel P. Borazanci E. H. Lee J. Amini A. Han I. W. (2020). Single-cell Transcriptome Analysis of Tumor and Stromal Compartments of Pancreatic Ductal Adenocarcinoma Primary Tumors and Metastatic Lesions. Genome Med. 12, 80. 10.1186/s13073-020-00776-9 32988401 PMC7523332

[B18] Lu S.-W. Pan H.-C. Hsu Y.-H. Chang K.-C. Wu L.-W. Chen W.-Y. (2020). IL-20 Antagonist Suppresses PD-L1 Expression and Prolongs Survival in Pancreatic Cancer Models. Nat. Commun. 11, 4611. 10.1038/s41467-020-18244-8 32929072 PMC7490368

[B19] Mace T. A. Shakya R. Pitarresi J. R. Swanson B. McQuinn C. W. Loftus S. (2018). IL-6 and PD-L1 Antibody Blockade Combination Therapy Reduces Tumour Progression in Murine Models of Pancreatic Cancer. Gut 67, 320–332. 10.1136/gutjnl-2016-311585 27797936 PMC5406266

[B20] Marco M. D. Grassi E. Durante S. Vecchiarelli S. Palloni A. Macchini M. (2016). State of the Art Biological Therapies in Pancreatic Cancer. Wjgo 8, 55–66. 10.4251/wjgo.v8.i1.55 26798437 PMC4714146

[B21] Olivier M. Hollstein M. Hainaut P. (2010). TP53 Mutations in Human Cancers: Origins, Consequences, and Clinical Use. Cold Spring Harbor Perspect. Biol. 2, a001008. 10.1101/cshperspect.a001008

[B22] O’Reilly E. M. Oh D.-Y. Dhani N. Renouf D. J. Lee M. A. Sun W. (2019). Durvalumab with or without Tremelimumab for Patients with Metastatic Pancreatic Ductal Adenocarcinoma. JAMA Oncol. 5, 1431–1438. 10.1001/jamaoncol.2019.1588 31318392 PMC6647002

[B23] Pattabiraman D. R. Weinberg R. A. (2014). Tackling the Cancer Stem Cells - what Challenges Do They Pose? Nat. Rev. Drug Discov. 13, 497–512. 10.1038/nrd4253 24981363 PMC4234172

[B24] Racle J. de Jonge K. Baumgaertner P. Speiser D. E. Gfeller D. (2017). Simultaneous Enumeration of Cancer and Immune Cell Types from Bulk Tumor Gene Expression Data. eLife 6. 10.7554/eLife.26476

[B25] Reimand J. Isserlin R. Voisin V. Kucera M. Tannus-Lopes C. Rostamianfar A. (2019). Pathway Enrichment Analysis and Visualization of Omics Data Using g:Profiler, GSEA, Cytoscape and EnrichmentMap. Nat. Protoc. 14, 482–517. 10.1038/s41596-018-0103-9 30664679 PMC6607905

[B26] Shi S. Cao H. (2014). Shikonin Promotes Autophagy in BXPC-3 Human Pancreatic Cancer Cells through the PI3K/Akt Signaling Pathway. Oncol. Lett. 8, 1087–1089. 10.3892/ol.2014.2293 25120662 PMC4114587

[B27] Siegel R. L. Miller K. D. Jemal A. (2018). Cancer Statistics, 2018. CA: A Cancer J. Clinicians 68, 7–30. 10.3322/caac.21442

[B28] Sung H. Ferlay J. Siegel R. L. Laversanne M. Soerjomataram I. Jemal A. (2021). Global Cancer Statistics 2020: GLOBOCAN Estimates of Incidence and Mortality Worldwide for 36 Cancers in 185 Countries. CA A. Cancer J. Clin. 71, 209–249. 10.3322/caac.21660

[B29] Thyagarajan A. Alshehri M. S. A. Miller K. L. R. Sherwin C. M. Travers J. B. Sahu R. P. (2019). Myeloid-Derived Suppressor Cells and Pancreatic Cancer: Implications in Novel Therapeutic Approaches. Cancers 11, 1627. 10.3390/cancers11111627

[B30] Waddell N. Pajic M. Pajic M. Patch A.-M. Chang D. K. Kassahn K. S. (2015). Whole Genomes Redefine the Mutational Landscape of Pancreatic Cancer. Nature 518, 495–501. 10.1038/nature14169 25719666 PMC4523082

[B31] Wang L. Xiong L. Wu Z. Miao X. Liu Z. Li D. (2018). Expression of UGP2 and CFL1 Expression Levels in Benign and Malignant Pancreatic Lesions and Their Clinicopathological Significance. World J. Surg. Onc 16, 11. 10.1186/s12957-018-1316-7

[B32] Yang Z. Li D. Liu Z. Miao X. Yang L. Zou Q. (2017). BIRC7 and KLF4 Expression in Benign and Malignant Lesions of Pancreas and Their Clinicopathological Significance. Cbm 17, 437–444. 10.3233/CBM-160660

[B33] Yi X.-P. Han T. Li Y.-X. Long X.-Y. Li W.-Z. (2015). Simultaneous Silencing of XIAP and Survivin Causes Partial Mesenchymal-Epithelial Transition of Human Pancreatic Cancer Cells via the PTEN/PI3K/Akt Pathway. Mol. Med. Rep. 12, 601–608. 10.3892/mmr.2015.3380 25707849

[B34] Zheng J. Huang X. Tan W. Yu D. Du Z. Chang J. (2016). Pancreatic Cancer Risk Variant in LINC00673 Creates a miR-1231 Binding Site and Interferes with PTPN11 Degradation. Nat. Genet. 48, 747–757. 10.1038/ng.3568 27213290

[B35] Zhong J. Jermusyk A. Wu L. Hoskins J. W. Collins I. Mocci E. (2020). A Transcriptome-wide Association Study Identifies Novel Candidate Susceptibility Genes for Pancreatic Cancer. J. Natl. Cancer Inst. 112, 1003–1012. 10.1093/jnci/djz246 31917448 PMC7566474

